# Exenatide Attenuates Obesity-Induced Mitochondrial Dysfunction by Activating SIRT1 in Renal Tubular Cells

**DOI:** 10.3389/fendo.2021.622737

**Published:** 2021-08-09

**Authors:** Yao Wang, Wei He, Wei Wei, Xiaoxue Mei, Ming Yang, Ying Wang

**Affiliations:** ^1^Department of Nephrology, The Affiliated Hospital of Yangzhou University, Yangzhou University, Yangzhou, China; ^2^Department of Endocrinology, The Affiliated Hospital of Yangzhou University, Yangzhou University, Yangzhou, China

**Keywords:** obesity, SIRT1, renal tubular cells, glucagon-like peptide-1 receptor agonist, mitochondrial dysfunction

## Abstract

Saturated free fatty acid (FFA)-induced lipotoxicity plays an important role in obesity-induced kidney injury. Exenatide, a Glucagon-like peptide-1 receptor agonist(GLP-1RA), protects against high-fat diet (HFD)-induced kidney injury. The precise mechanism needs to be further explored. This study investigated whether exenatide protects against FFA-induced tubular epithelial cells (TECs) lipotoxicity and elucidated its underlying mechanisms. Here, we show that exenatide treatment reversed HFD induced TECs injuries, including TECs apoptosis and SIRT1 downregulation. The efficacy of exenatide was better than simvastatin. In palmitate (PA)-stimulated HK2 cells, exenatide treatment reversed the downregulation of SIRT1 and prevented an increase in reactive oxygen species (ROS) production, a decrease in mitochondrial membrane potential, and mitochondrial apoptosis. The renal-protective effects of exenatide on the generation of mitochondrial ROS and mitochondrial apoptosis were blocked by inhibiting SIRT1 activation. Collectively, these findings show that exenatide was superior to simvastatin in the treatment of obesity-TECs injuries, the mechanism is partially through SIRT1 restoration, which directly reverses mitochondrial dysfunction and apoptosis.

## Introduction

Obesity has emerged as an escalating public health problem, resulting in shortened life expectancy, lowered quality of life and increased medical expenditure (1). In recent years, a close association between obesity and renal dysfunction has become apparent. Additionally, epidemiologic analyses have shown that obesity is an independent risk factor for chronic kidney disease (CKD) (2). Obesity is characterized by elevated circulating free fatty acids (FFAs) and hypertriglyceridemia. Elevated FFAs and deleterious FFA‐derived lipids lead to metabolic disorders, cellular dysfunction, and cell death in nonadipose tissues, which is known as lipotoxicity (3). Numerous studies have indicated that the overaccumulation of FFAs in the kidney induces podocyte, proximal tubular epithelial cells (TECs) and tubulointerstitial tissue damage through different mechanisms, particularly by boosting the production of reactive oxygen species (ROS) and promoting mitochondrial damage and tissue inflammation (4).

Glucagon-like peptide-1 (GLP-1) is an intestinal peptide hormone that is secreted by L cells. GLP-1 and the GLP-1 receptor (GLP-1R) regulate insulin synthesis, appetite, and weight loss and promote glucose uptake. GLP-1R agonist (GLP-1RA) has been clinically applied in the treatment of type 2 diabetes and obesity (5). Moreover, recent clinical trials have demonstrated markedly good renal outcomes among available GLP-1RAs (6), which were recommended by the American and European Diabetes Association as a priority for diabetic patients with kidney disease in 2019 (7). The benefits might be consistent with the known effects of GLP-1RA on traditional risk factors for progressive kidney disease, including improving glucose metabolism, lowering blood pressure and reducing body weight (8). Emerging evidence suggests that GLP-1 RA has multiple effects in the kidney, including inhibiting NHE3-dependent sodium reabsorption (9), RAAS overactivation (10), inflammation and neural signaling (11). Interestingly, exenatide, a GLP-1RA, has a remarkable weight loss effect in obesity (12)and acts in a SIRT1-dependent manner (13).

SIRT1, an NAD^+^-dependent class III histone deacetylase, has been demonstrated to play numerous roles in cellular processes and whole-body metabolisms, such as in mitochondrial function, energy homeostasis, longevity, and apoptosis (14). Generally, SIRT1 is expressed in the renal cortex and medulla (15), and both SIRT1 overexpression and SRT1720 (a SIRT1 activator) treatment reduce renal lipid contents and decrease the expression of lipogenesis and oxidative stress in high-fat diet (HFD)-fed mouse offspring (16).Exenatide has been shown to prevent apoptosis in HFD-induced renal tubular injury (17). However, whether the renoprotective roles of exenatide are due to the restoration of SIRT1 remains unclear.

In light of the above considerations, the roles of exenatide in palmitate (PA)-treated HK2 renal TECs and HFD-fed C57BL/6 mice were investigated in TECs both *in vitro* and *in vivo.* We especially focused on the changes of SIRT1 and the renal outcomes. Here, we show that in response to exenatide treatment, the level of SIRT1 is restored, thus leading to the reversal of mitochondrial dysfunction, including ROS accumulation and apoptosis.

## Methods

### Animals

All animal procedures were reviewed and approved by the Institutional Animal Care and Use Committee at Yangzhou University (China). Six-week-old male C57BL/6 mice were obtained from the Experimental Animal Center of Yangzhou University and were housed on a 12-h light-dark cycle. All mice were housed in specific pathogen-free (SPF) facilities. The mice were divided into four groups (n = 10 per group) as follows: the control with standard diet (SD)group; HFD (HFD)group; HFD with exenatide treatment (HFD+E)group; and HFD with simvastatin treatment (HFD+S)group as a positive control of lipid-lowering therapy. The mice had free access to standard rodent chow (10% of total calories from fat) or HFD (60% of total calories from fat) and water for 12 weeks. Starting at 6 weeks, exenatide was subcutaneously injected (Baxter 0.12 mg/kg/d) and simvastatin (Merck 20 mg/kg/d) was diluted in DMSO and intra-gastrically administered (18). The HFD group received normal saline alone. Before weight the bodyweight and tested the blood glucose, the mice were fasted regularly overnight for 12h every week. At the end of the 12 week treatment period, all mice were sacrificed after 12h fasted. Orbital venous blood samples were obtained. The blood glucose were measures with IELTS Blood Glucose Analyzer. The blood samples was collected in the collector containing EDTA anticoagulant low density lipoprotein cholesterol. After cooling on ice for 30 min, the plasma was separated at 4000r/min, 20mins, 4°C. Serum levels of Total cholesterol (TC) and Triglyceride (TG) were detected by Beckman Coulter AU 480. Kidneys were perfused with saline *via* the abdominal aorta and were then immediately harvested. Part of the tissues was fixed for histological analysis, and the remaining tissues were stored at -80°C for further investigations.

### Histological Examination

Kidney tissues were fixed with 4% paraformaldehyde, paraffin-embedded and then cut with a microtome. Paraffin-embedded tissue was dewaxed, dehydrated and stained with hematoxylin and eosin (H&E), periodic acid-Schiff (PAS) and Masson’s trichrome, followed by histological examination. The histopathological scoring analysis of the kidney was evaluated by two independent pathologists who were blinded to the group information. Histopathological changes, including loss of brush border, tubular dilation, tubule vacuolar degeneration, and cell lysis, were evaluated. Tissue damage was examined using the tubular injury score(TIS) as previously described: 0 for no damage; 1 for <25%; 2 for 25~50%; 3 for 50~75% and 4 for >75% (19).

### Immunohistochemistry

Briefly, for the immunohistochemical staining of SIRT1, tissue sections were boiled in EDTA antigen retrieval buffer. After cooling, sections were incubated to block endogenous peroxidase activity with 3% hydrogen peroxide solution at room temperature for 15 min. Sections were then incubated overnight at 4°C in a humid chamber with an antibody against SIRT1 (1:200 dilution, GT1189,Genetex, China). Then, tissue sections were incubated with a biotinylated secondary antibody for 1 h. Finally, sections were counterstained with hematoxylin.

### Cell Culture

An immortalized human proximal tubular cell line (HK2) was cultured in 100-mm dishes. HK2 cells were cultured in DMEM/F12 supplemented with 10% fetal bovine serum in an atmosphere of 5% CO_2_ at 37°C. HK2 cells from different groups were plated in 6-well plates (8×10^5^ per well) and 12-well plates (3.5×10^5^ per well) before treating. PA was prepared by conjugation of PA with BSA (Sigma-Aldrich). Sodium palmitate (Sigma-Aldrich) were dissolved in 100% ethanol at 70-degree centigrade, and then vigorously mixed with 0.5% BSA in PBS at a molar ratio of 10:1 at 50-degree centigrade, filter-sterilized, and added to culture media at the final PA concentration of 0.5mM. HK2 cells were starved overnight in culture medium without fetal bovine serum prior to treatment with PA, exenatide (100 nM) or Selisistat (10 µM) for 24 h (20). HK2 cells with 0.5% BSA served as controls. HK2 cells were fixed with 4% paraformaldehyde for TdT-mediated dUTP nick-end labeling (TUNEL) assay or lysed with RIPA buffer and a protease inhibitor cocktail for Western blotting. Protein expression levels of SIRT1, KIM-1,peroxisome proliferator-activated receptor-γ co-activator 1α (PGC-1α), Bax, and cleaved caspase-3 were measured by immunoblotting assays. Apoptotic nuclei were detected using a TUNEL kit to measure apoptosis. Cell-Counting Kit-8 (CCK8) (Dojindo Molecular Technologies, Inc., Tokyo, Japan), crystal violet staining and a cytotoxicity LDH assay kit (Wuhan Beyotime Biotechnology Co. Ltd., China) were used to assay cell viability, and the change in the mitochondrial membrane potential (ΔΨm) were detected using JC-1. All procedures were performed following the manufacturer’s instructions.

### Western Blotting

Western blotting was performed as previously described (21). Mouse kidney and cultured cells were lysed in RIPA lysis buffer. Anti-GAPDH (1:2000 dilution, CW0100M, CWBio, China), anti-cleaved caspase 3 (1:1000 dilution, ab32503, Abcam, Cambridge, UK), anti-Bcl2 (1:1000 dilution, cst2870, Cell Signaling Technology, USA), anti-SIRT1 (1:1000 dilution, GT1189, Genetex, China),anti-KIM-1(0.25ug/ml, AF1817, RD systems, Canada), anti-PGC-1a(1:1000, # 2178, cell signaling technology, USA) and anti-Bax (1:1000 dilution, ab193349, Abcam Cambridge, UK) antibodies were used in this study. Experiments were repeated three times.

### Mitochondrial Membrane Potential (ΔΨm)

ΔΨm was determined by the cationic fluorescent dye JC-1. JC-1 showed two distinct subpopulations with different ΔΨm values: red with high ΔΨm and green with low ΔΨm. This shift of JC-1 fluorescence from red to green indicated a collapse of ΔΨm. Briefly, HK-2 cells were fixed with a working solution of JC-1 (1 mg/ml) in a 5% CO_2_ incubator at 37°C for 20 min and were then washed twice with incubation buffer. The fluorescence of JC-1 was recorded with a Leica fluorescence microscope. The red or green fluorescence intensity and the ratio of red/green were calculated and analyzed by NIH ImageJ software (National Institutes of Health, Bethesda, MD). The level of ΔΨm was reflected by the ratio of red/green.

### Statistical Analysis

Statistical analysis was performed with GraphPad Prism 6 software (GraphPad, San Diego, CA, USA), and data are presented as the mean ± standard deviation (SD). Statistical differences between groups were analyzed by one-way ANOVA. Tukey-Kramer test were used as a post-hoc test after ANOVA for all results. A two‐sided *P*  < 0.05 was considered significant.

## Results

### Exenatide Alleviates HFD-Induced Weight Gain, Dyslipidemia and Renal Tubular Injury in Mice

**Figure 1:**
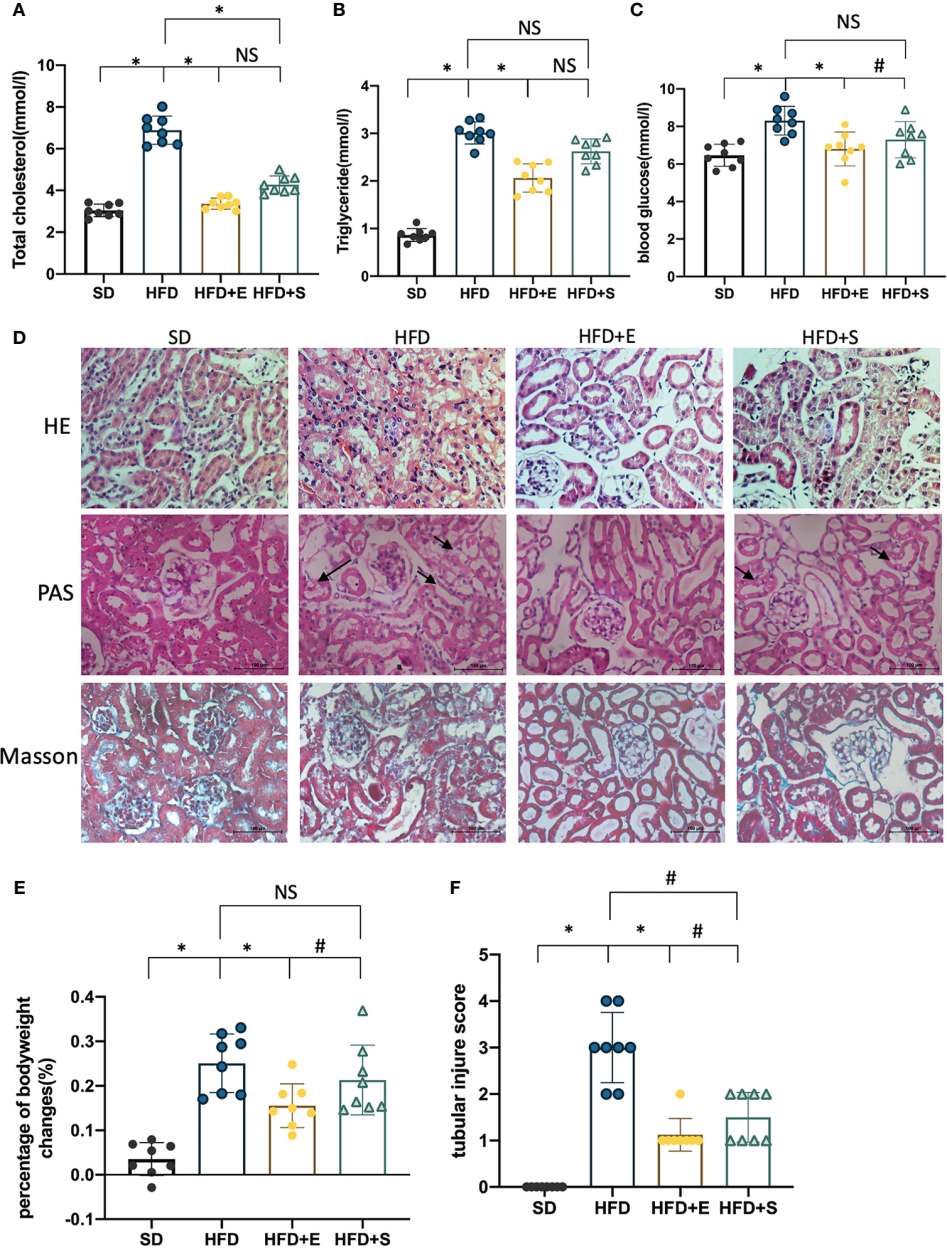
Exenatide improves HFD-induced weight gain, dyslipidemia and renal tubular injury in mice. **(A–C, E)** Effects of exenatide and simvastatin on TC, TG, blood glucose and body weight changes in HFD mice. **(D)** H&E, PAS and Masson-trichrome staining of the kidney, arrowheads indicate vacuolation changes in TECs (original image magnification: ×200, bars =100 μm). **(F)** Quantification of tubular injury. Data are presented as the mean ± SD (n=8). SD, standard diet; HFD, high-fat diet; HFD+E, HFD with exenatide treatment; HFD+S, HFD with simvastatin treatment. *P < 0.01 *versus* HFD group. ^#^p < 0.01 versus HFD+E group. NS no statistical difference between groups.

To investigate the metabolic effects of exenatide *in vivo*, HFD mice were treated with exenatide, and simvastatin was used as a lipid-lowering control. The results suggested that both exenatide (0.12 mg/kg/d) and simvastatin (20 mg/kg/d) significantly improved lipid metabolism by reducing TC (**Figure 1A**). However, only exenatide could lower serum TG, blood glucose and HFD-elevated body weight (**Figures 1B, C, E**). In addition, HE, PAS and Masson’s trichrome staining were carried out to investigate the roles of exenatide on the histology of renal tissues in HFD mice. The most significant pathological change in HFD groups is the vacuolation of the TECs, as indicated by the arrowheads. There were also some tubular dilation and cell lysis. Exenatide treatment reversed those changes. However, treated with simvastatin only partly reverse TECs dilation and vacuolation. Those changes were accessed by TIS, which were significantly elevated in the HFD groups, whereas were ameliorated after treatment with exenatide or simvastatin (**Figure 1F**). There were no significant fibrosis changes in all groups (**Figure 1D**). Therefore, exenatide can decrease body weight, ameliorate dyslipidemia, and especially attenuate renal tubular injury in HFD mice.

### Exenatide Attenuates HFD-Induced Renal Tubular Cells Apoptosis in Mice

**Figure 2:**
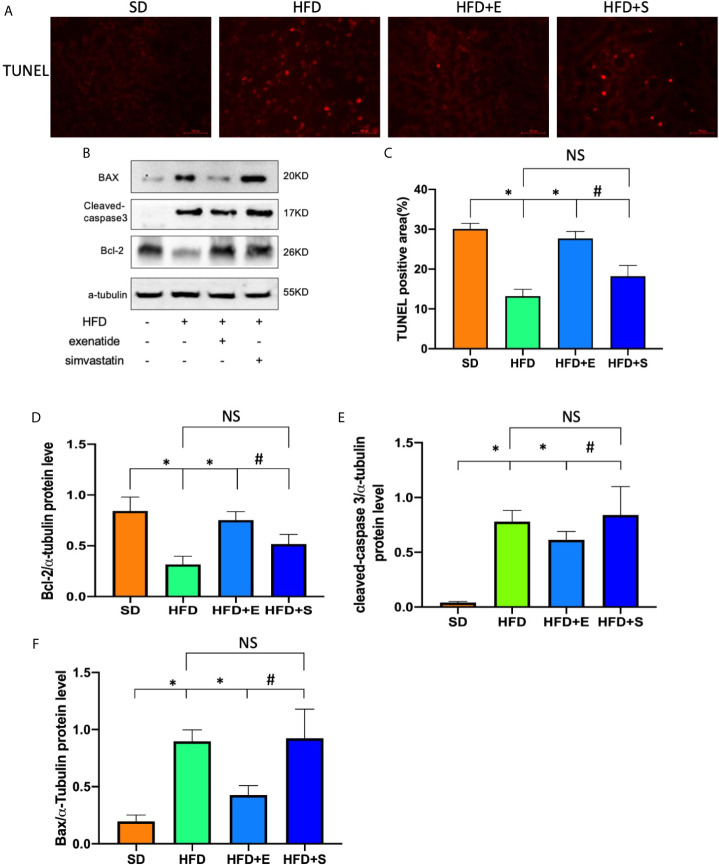
Exenatide attenuated renal tubular apoptosis in HFD mice. **(A)** Kidney apoptosis was detected by TUNEL staining and fluorescence microscopy (original image magnification: ×200, bars=100μm). **(B)** Western blots of cleaved caspase-3, BAX and Bcl-2 in renal lysates. **(C)** Quantification of TUNEL-positive cells in the kidney. **(D–F)** Relative protein expression of cleaved caspase-3, bax and Bcl-2. α-tubulin was used as a control for protein loading. Data are presented as the mean ± SD (n=3). *P < 0.01 versus HFD group. ^#^p < 0.01 versus HFD+E group, NS no statistical difference between groups.

To test the effects of exenatide on tubular apoptosis in mice fed an HFD, we performed a TUNEL assay and Western blot analysis of bax, cleaved caspase-3 and Bcl-2. The results showed that an HFD induced renal cell apoptosis, which could be reversed after treatment with exenatide (**Figures 2A, B**). In addition, the protein levels in the HFD group showed a strong increase in bax, cleaved caspase-3 and a decrease in Bcl-2 (**Figures 2C–F**). Interestingly, combinates treatment with HFD and exenatide, but not simvastatin, significantly reversed these protein expression changes. Altogether, these results suggest that HFD induces renal tubular apoptosis, while exenatide can potentially attenuate it. As simvastatin treatment did not ameliorate HFD-induced apoptosis, we proposed that the anti-apoptosis effect of exenatide in TECs is independent of amelioration of hyperlipidemia.

### Exenatide Restores HFD-Inhibited SIRT1 Expression and TECs Injury in Mice

**Figure 3:**
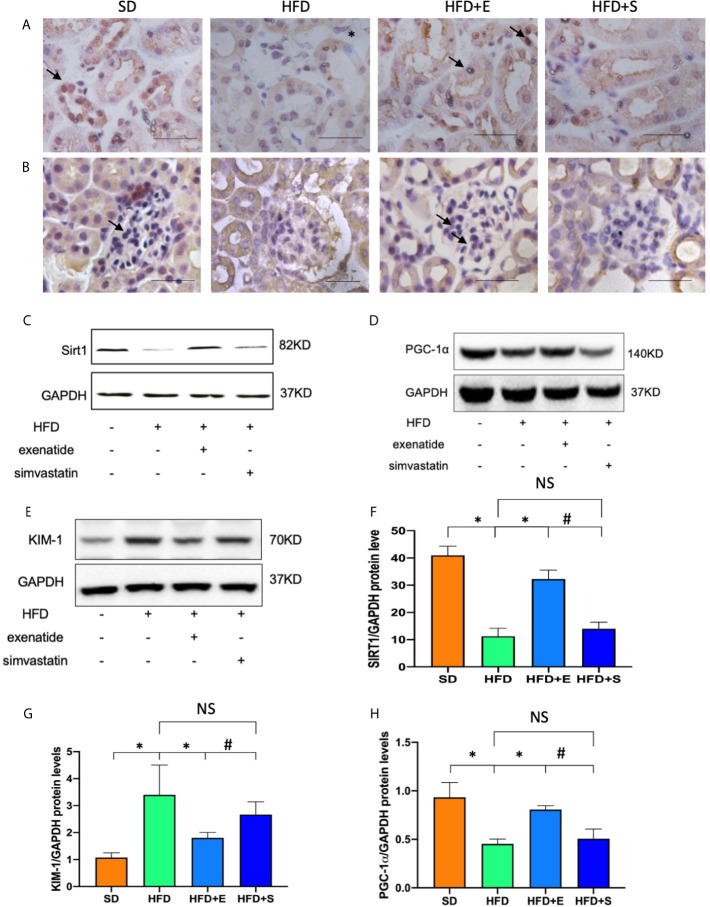
Exenatide restored HFD-repressed SIRT1 expression in mice. **(A, B)** Immunohistochemistry analysis of SIRT1 expression in kidney tissue (original image magnification: × 200, bars= 100 μm). **(C)** Western blots of SIRT1 in renal lysates. **(D)** Western blots of PGC-1α in renal lysates. **(E)** Western blots of KIM-1 in renal lysates. **(F–H)** Relative protein expression of SIRT1, PGC-1α, KIM-1. GAPDH was used as a control for protein loading. Data are presented as the mean ± SD (n=3). *P < 0.01 *versus* HFD group. ^#^p < 0.01 *versus* HFD+E group. NS no statistical difference between groups.

Exenatide is known to improve diabetes through SIRT1 restoration. we were interested in whether the protection role of exenatide is related to the SIRT1 changes in the kidney in obesity. We tested the SIRT1, and its downstream factor PGC-1α protein changes in the four groups of mice. SIRT1 immunohistochemistry staining of renal tissues from control and HFD mice was performed. As shown in **Figures 3A, B**, there was SIRT1 stain in the nuclear of renal cell, including renal tubular cells and glomeruli; HFD mice showed lower SIRT1 expression levels than the control group, while exenatide, but not simvastatin, treatment significantly reversed this reduction. In addition, the immunoblot results for SIRT1 and its downstream factor PGC-1α showed a similar trend as the immunohistochemistry analysis (**Figures 3C, D, F, H**). As we had observed obvious pathological changes in TECs in HFD-mice, and exenatide treatment had a better outcome in TECs than simvastatin, we further tested KIM-1 protein, the renal TECs injury marker, the result indicated that exenatide, but not simvastatin, ameliorated the HFD-mediated TECs injury (**Figures 3E, G**), In summary, our findings suggest that exenatide prevents the HFD-mediated TECs injury, concomitantly restore SIRT1 and PGC-1α expression.

### Exenatide Suppresses PA-Induced Apoptosis *via* SIRT1 Restoration

**Figure 4:**
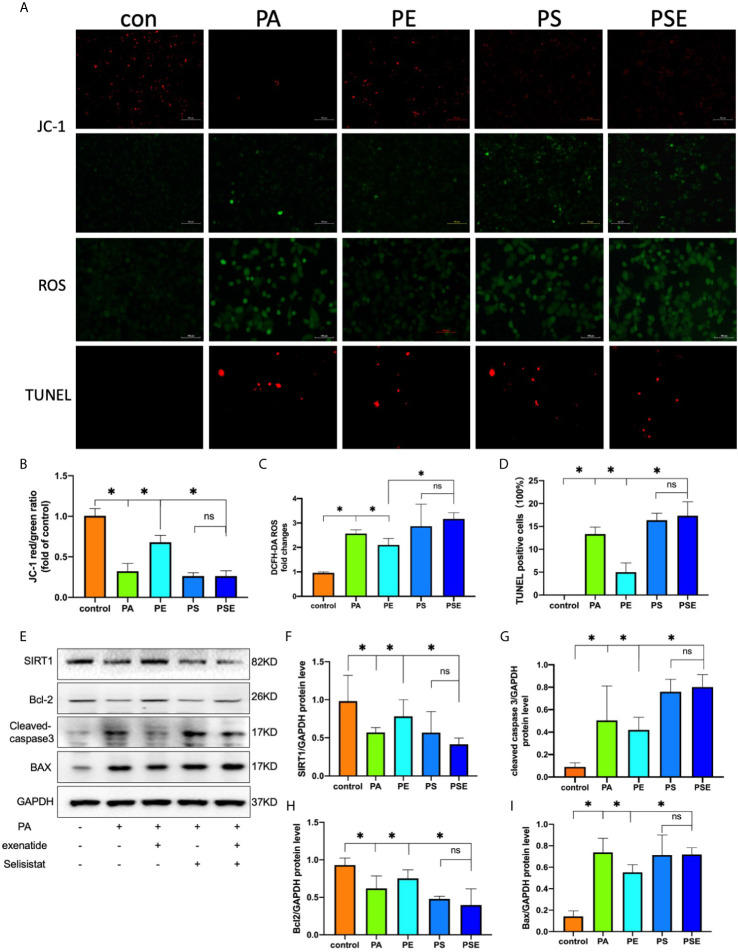
Exenatide suppresses PA-induced apoptosis *via* SIRT1 restoration. HK2 cells were treated with 100 nM exenatide plus 10 µM Selisistat and incubated with PA (0.5 mM) for 24 h. **(A)** Intracellular mitochondrial membrane potential was examined by JC-1 staining, intracellular ROS was examined by DCFH-DA staining and apoptosis was examined by TUNEL staining, all of which were detected by fluorescence microscopy (original image magnification: ×200, bars=100 μm). **(B–D)** Calculated mean fluorescence intensity of JC-1 and DCFH-DA staining and mean percentages of TUNEL staining-positive cells. **(E)** Western blots of SIRT1, cleaved caspase-3, Bcl-2 and Bax in cell lysates. **(F–I)** Relative protein expression of SIRT1, cleaved caspase-3, Bcl-2 and Bax. GAPDH was used as a control for protein loading. CON, control; PA, palmitate; PE, treat with PA and exenatide (100 nM); PS, treat with PA and Selisistat (10 µM); PSE, treat with PA, exenatide (100 nM) and Selisistat (10 µM). Data are presented as the mean ± SD (n=3). *P < 0.01 *versus* PA group. NS no statistical difference between groups.

The above results provided evidence that exenatide improves HFD-induced renal tubule injury and SIRT1 expression. These findings motivated us to investigate whether SIRT1 activation directly mediates the actions of exenatide against PA-induced renal tubule injury. The SIRT1 inhibitor selisistat was applied in this study to repress SIRT1 activation in HK-2 cells. The results showed that SIRT1 inhibition decreased the protective effect of exenatide against PA-reduced mitochondrial dysfunction, as indicated by increased ROS production (**Figures 4A, C**), decreased ΔΨm (**Figures 4A, B**) and increased apoptosis(**Figures 4A, D**). In addition, exenatide decreased mitochondrial apoptosis, as indicated by cleaved caspase-3 and Bax activation and Bcl-2 inhibition. These effects were abolished by the inhibition of SIRT1 with selisistat (**Figures 4E–I**). These data indicate that exenatide treatment prevents mitochondrial dysfunction in a SIRT1-dependent manner.

## Discussion

**Figure 5:**
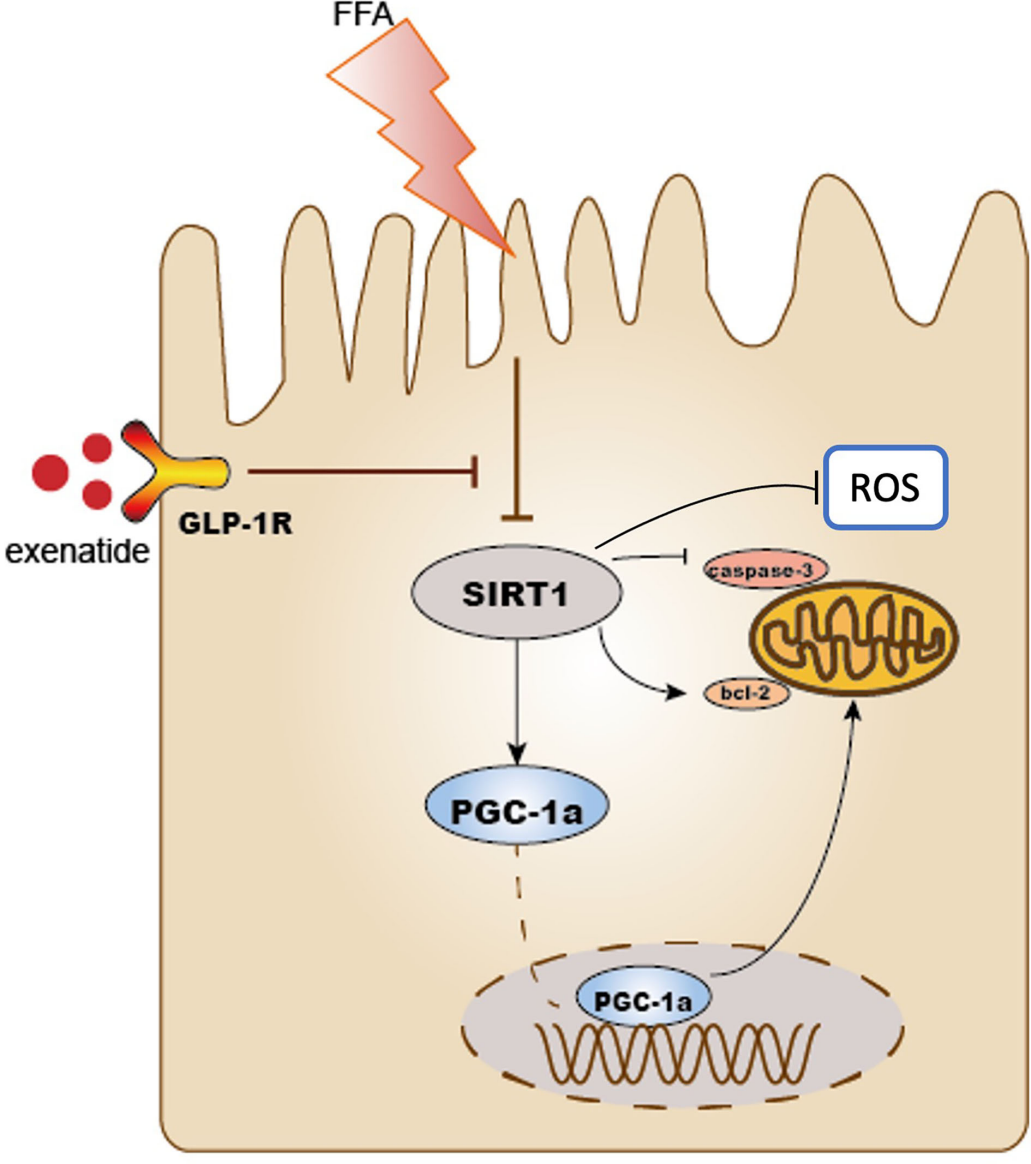
Schematic diagram of this study. Exenatide protects HFD induced tubular injury through the preservation of SIRT1. SIRT1 positively regulates the expression of PGC-1α. HFD reduced the expression of SIRT1 and PGC-1α subsequently leads to mitochondria-derived ROS production and mitochondrial apoptosis, which results in the development of renal tubular injury. Treatment with exenatide increased the expression of SIRT1 and PGC-1α and then prevented obesity-induced mitochondrial dysfunction. As a result, exenatide suppressed mitochondria-derived ROS production, alleviated mitochondrial dysfunction, reduced cell apoptosis, and protected against HFD-induced renal tubular injury.

Obesity can directly lead to kidney damage and is a major risk factor for the development of CKD (22), even independent of its association with hypertension, diabetes, and dyslipidemia (23). Our data revealed that exenatide can significantly improve the elevation of blood glucose, plasma lipid profile and reduce weight gain caused by HFD. It can also significantly improve TECs injury, including TECs vacuolation and apoptosis. Interestingly, the renal-protection effect is better than that of lipid-lowering medicine simvastatin. Our results suggest that exenatide prevented TECs mitochondrial dysfunction in HFD mice *via* activating SIRT1-PGC-1α signalling. Activation of SIRT1 alleviated mitochondrial dysfunction, suppressed mitochondrial ROS production, reduced cell apoptosis, and consequently improved TECs injury (**Figure 5**). Our data demonstrate for the first time that exenatide prevents against TECs mitochondrial dysfunction in a SIRT1-dependent manner.

Tubular apoptosis plays an important role in the progression of nephropathy. In this study, we found that exenatide ameliorated TECs apoptosis both *in vitro* and *in vivo.* Then, the underlying mechanism of the antiapoptotic properties of exenatide was investigated. TECs are rich in mitochondria because they have limited glycolytic capability. Mitochondria play a central role in energy metabolism. Some studies have revealed that FFAs modulate mitochondrial ROS production in obesity *via* several mechanisms, including interacting with components of the respiratory chain, thereby inhibiting electron transport (24) and autophagy (25). Mechanistically, FFAs provoke cell death and converge on the mitochondria through the activation of the intrinsic apoptotic pathway, including the inhibition of Bcl-2, release of cytochrome c, and subsequent induction of the cleavage and maturation of caspase 3 (26). Mitochondrial apoptosis induced by FFAs has been reported in adipocytes, endothelial cells and stem cells (27). In the kidney, HFD increases mitochondrial ROS (28) and induces mitochondrial apoptosis in tubular cells (29). Consistent with previous studies, we found that the exposure of HK2 cells to PA induced mitochondrial membrane potential changes, ROS production and apoptosis, and treatment with exenatide mitigated PA-induced mitochondrial dysfunction. These results suggest that the beneficial effects of exenatide on reducing apoptosis by ameliorating mitochondrial dysfunction.

Renoprotective effects of GLP-1Rs were consistently reported in preclinical and clinical studies (30, 31). It had been reported that GLP-1 constructs significantly reduced tubulointerstitial renal damage, and attenuated renal accumulation of macrophages and T cells (32). In our study, exenatide mitigated PA-induced TECs mitochondrial dysfunction and apoptosis, concomitantly with SIRT1 restoration. Several studies have demonstrated that SIRT1 mediates the effect of exenatide on lipid homeostasis (13, 33). SIRT1 exerts cytoprotective effects by regulating mitochondrial dynamics (34). In the kidney, SIRT1 is widely expressed in tubular cells and podocytes (35). The expression of SIRT1 has reduced in diabetes milieus, obesity (36) and during aging (37). The decreased level of SIRT1 is associated with an increased level of apoptosis after unilateral ureteral obstruction(UUO), and a SIRT1 activator reduced this kind of damage (38). It has been reported that exenatide reduced fat mass and enhanced the lipolytic in a SIRT1-dependent manner (13), so we were interested in whether the renal-protection role of exenatide is associated with SIRT1 activation. Moreover, PGC-1α is a substrate of SIRT1, which deacetylates PGC-1α and thus improves the transcriptional activity of PGC-1α (39). PGC1-α balances mitochondrial dynamics and energetics to maintain mitochondrial homeostasis (40). Based on our data, exenatide treatment reversed the decreased protein level of SIRT1 and PGC-1α, concomitantly ameliorating ROS production, decreased mitochondrial membrane potential, and mitochondrial apoptosis in TECs both *in vitro* and *in vivo*. These results suggest that the renal protective effect of exenatide may be related to the activation of SIRT1.

Furthermore, we investigated whether exenatide could be protective when SIRT1 was inhibited. The results showed that exenatide did not exert a protective role against PA-induced apoptosis in HK2 cells when SIRT1 activation was inhibited. This finding could be the result of exenatide ameliorating HK2 cell apoptosis in a SIRT1-dependent manner. Our study presents a new mechanism for GLP1-RA treatment in obese kidneys: namely, the GLP1-RA exenatide can rescue SIRT1, which can ameliorate obesity-induced mitochondrial dysfunction, the overproduction of ROS and apoptosis.

There is still some limitation in our study, we did not observe any difference in renal function indexes, including serum urea and creatinine (not presented), both in HFD and SD groups, the renal fibrosis was not significant. On the one hand, the renal injury process of HFD is relatively long. We only observed for 12 weeks, which is not long enough to form serious renal damage. We will further extend the observation window to systematically evaluate the renal protective effect of exenatide. Secondly, changes in urea and creatinine are rather indexes of glomerular filtration rate changes than early tubular injuries. We had evaluated the tubular damage marker KIM-1 to further clarify the protective effect of exenatide on TECs.

In summary, our study demonstrates that HFD results in TECs impair by triggering SIRT1 downregulation mediated mitochondrial dysfunction. Exenatide prevents mitochondrial dysfunction and improves TECs injury in mice with HFD through the SIRT1-PGC1α pathway, which negatively regulates the expression of ROS and mitochondrial apoptosis. These findings identify exenatide-modulated SIRT1 restoration as a potential target for treating TECs mitochondrial dysfunction in obesity.

## Data Availability Statement

The raw data supporting the conclusions of this article will be made available by the authors, without undue reservation.

## Ethics Statement

The animal study was reviewed and approved by the Institutional Animal Care and Use Committee at Yangzhou University (China).

## Author Contributions

YaW and YiW designed the study. YaW, XM, and WH carried out the experiments. WW and YaW analyzed experimental results. YaW and MY completed the manuscript. All authors contributed to the article and approved the submitted version.

## Funding

This work was supported by the Youth fund of National Natural Science Foundation of China No. 81200596, the Science and Technology Research Programs of Health and Family Planning Commission of Jiangsu Province No. Z2017018, and the Social Development Program of Yangzhou Science and Technology Bureau No. YZ2020276.

## Conflict of Interest

The authors declare that the research was conducted in the absence of any commercial or financial relationships that could be construed as a potential conflict of interest.

## Publisher’s Note

All claims expressed in this article are solely those of the authors and do not necessarily represent those of their affiliated organizations, or those of the publisher, the editors and the reviewers. Any product that may be evaluated in this article, or claim that may be made by its manufacturer, is not guaranteed or endorsed by the publisher.
