# Supplementary figures and images for: Exenatide Attenuates Obesity-Induced Mitochondrial Dysfunction by Activating SIRT1 in Renal Tubular Cells
Source: Front Endocrinol (Lausanne). 2021 Aug 9;12:622737. doi: 10.3389/fendo.2021.622737 (PMC8380782; doi:10.3389/fendo.2021.622737)

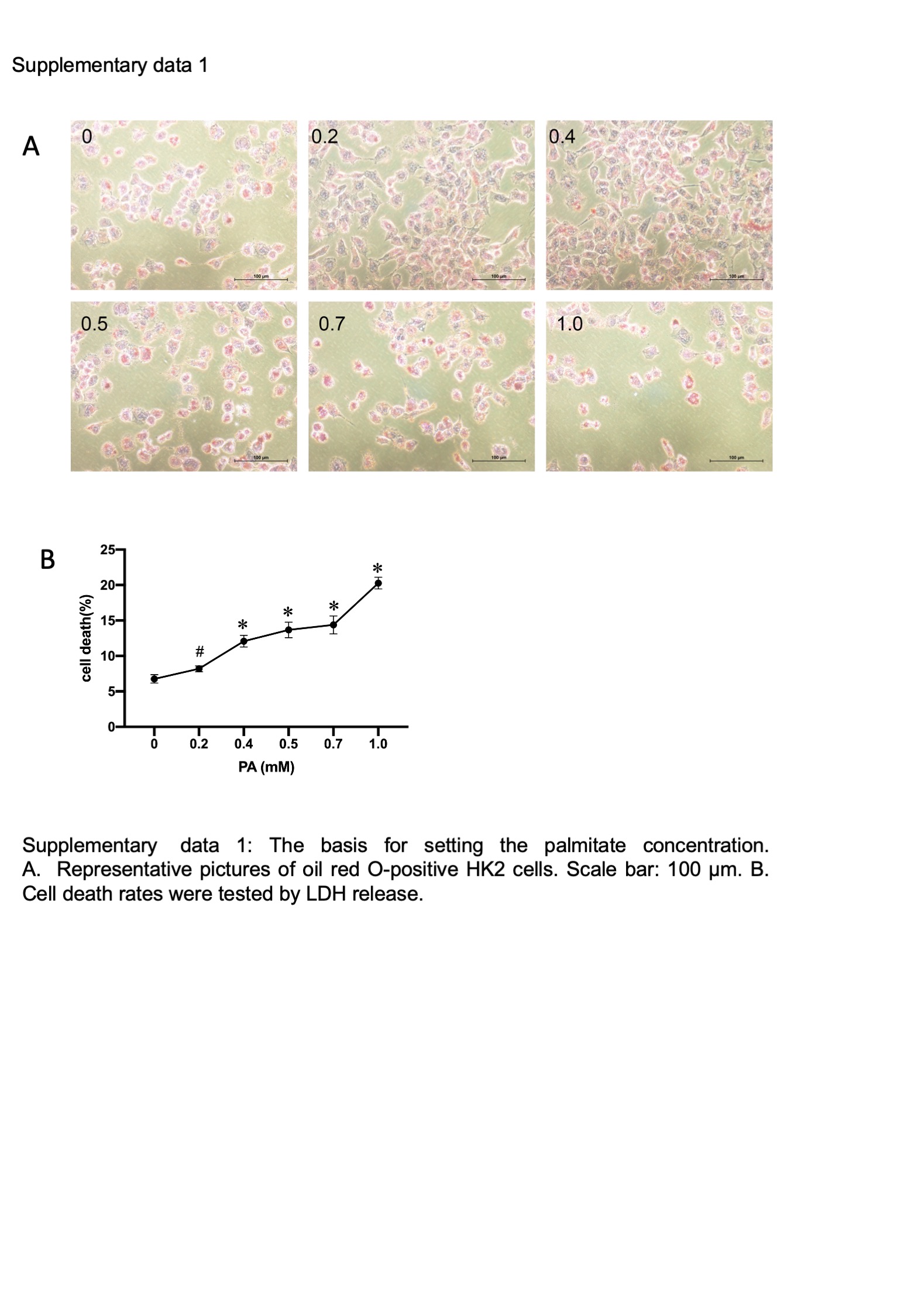

Supplement: Supplementary file 1 [file Image_1.jpeg]
